# Clinical Anthropometry in Male Infertility: Differences Between Men With and Without Klinefelter Syndrome

**DOI:** 10.7759/cureus.98793

**Published:** 2025-12-09

**Authors:** Ryo Sato, Takayuki Sugiyama, Rikiya Matsumoto, Hiroshi Terada, Teruo Inamoto

**Affiliations:** 1 Department of Urology, Chutoen General Medical Center, Kakegawa, JPN; 2 Department of Urology, Hamamatsu University School of Medicine, Hamamatsu, JPN

**Keywords:** azoospermia, klinefelter's syndrome, male factor infertility, male infertility, oligospermia

## Abstract

Introduction: Klinefelter syndrome (KS) is one of the most common genetic causes of male infertility and is frequently associated with characteristic anthropometric features. However, few studies have directly compared body proportions between patients with KS and infertile men without KS in clinical settings in Japan. Therefore, the present study investigated physical characteristics, particularly arm span, in infertile men with or without KS.

Patients and Methods: The present study included 30 infertile Japanese men with KS and 289 infertile men without KS who visited the outpatient clinic for male infertility at our institution between January 2004 and July 2025. Clinical data included age, height, weight, body mass index (BMI), and arm span.

Results: Arm span was significantly longer in the KS group than in the non-KS group (p = 0.046). Height (p = 0.019) and weight (p = 0.017) were also significantly higher in the KS group, whereas BMI did not significantly differ between the groups.

Conclusion: Infertile Japanese men with KS had a significantly longer arm span than non-KS infertile men, consistent with eunuchoid body proportions. These results underscore the clinical value of incorporating a routine anthropometric assessment, particularly an arm span measurement, in the clinical evaluation of male infertility.

## Introduction

Infertility affects approximately 8-12% of couples worldwide, and male factors are responsible for nearly half of these cases [1,2]. Azoospermia, defined as the complete absence of sperm in the ejaculate, is identified in approximately 1% of all men and 10-15% of infertile males [3]. Region-specific estimates show notable variation: male-factor infertility is reported to account for approximately 2.0-3.1% of infertility cases in Central and East Asia, and male involvement in infertility reaches about 37% across Asia as a whole [2]. Therefore, a comprehensive understanding of the genetic and endocrine causes of male infertility is essential for an accurate diagnosis and appropriate counselling.

Among the genetic causes, Klinefelter syndrome (KS) is the most common sex chromosome abnormality in males and a major contributor to male infertility [4]. The prevalence of KS is approximately 1 in 500-1,000 live male births, accounting for 10-12% of men with azoospermia [4]. Despite this, many cases remain undiagnosed until adulthood [5]. This underdiagnosis persists because the clinical presentation of KS is often subtle and heterogeneous during childhood and adolescence. Many patients exhibit only mild features, such as tall stature, small testes, or variable degrees of androgen deficiency, which do not prompt early medical evaluation. These nonspecific and age-dependent manifestations frequently lead to delayed recognition, with many cases diagnosed only during infertility workup in adulthood [4,5].

One of the most recognisable somatic features of KS is the presence of eunuchoid body proportions, typically defined by an increased arm span relative to height and a low upper/lower segment ratios [4]. Large clinical cohorts of infertile men with KS demonstrated that patients often have a greater arm span relative to height, reflecting androgen deficiency-related disproportionate limb growth [6]. In addition, population-based datasets confirmed that patients with KS were generally taller with longer arm spans than non-KS controls [7]. These anthropometric differences have a clear biological basis: androgen deficiency during puberty delays epiphyseal closure, allowing continued longitudinal growth of the long bones and resulting in disproportionate limb elongation [4].

Nevertheless, few studies have directly compared anthropometric data between KS and non-KS infertile men within the same clinical context, and easily measurable traits, such as arm span, remain underutilised in daily practice. Outcome reporting in KS research has also been inconsistent, with heterogeneous definitions of both anthropometric and reproductive parameters, emphasising the need for standardised core outcome sets [8]. Accordingly, the present study investigated physical characteristics-particularly arm span-in infertile men with and without KS in a Japanese hospital-based cohort.

## Materials and methods

Ethics

The present study was conducted in accordance with the guidelines of the Declaration of Helsinki and was approved by the Research Ethics Committee at our institution (No. 1331251030). The need to obtain informed consent from patients was waived because of its retrospective design; however, an opportunity to opt out of this study was provided through the institutional website.

Study design and population

The retrospective cross-sectional study included men who visited the outpatient clinic for male infertility at our institution between January 2004 and July 2025. All patients underwent a physical examination and hormonal assessment as part of the routine diagnostic work-up for infertility. In addition, all men with abnormal semen parameters routinely underwent karyotype analysis according to our institutional protocol. Patients with genetically confirmed KS (47, XXY or mosaic 46, XY/47, XXY) were assigned to the KS group (n = 30; 27 non-mosaic, 3 mosaic). Infertile men without KS served as the non-KS group (n = 289). Exclusion criteria included a history of malignancy or systemic disease that may affect the hormonal status or body composition, severe skeletal deformity that may affect anthropometric measurements, prior hormonal therapy at another institution, or incomplete essential data.

Anthropometric measurements

Age, height, weight, and arm span (fingertip-to-fingertip distance with arms extended horizontally) were recorded at the first visit. Body mass index (BMI) was calculated as weight (kg) divided by height squared (m²). All anthropometric measurements were performed by trained nursing staff using standardised protocols at our institution. Height and weight were measured with calibrated clinic stadiometers and scales, respectively, and arm span was assessed using a standardised wall-mounted measuring tape with patients positioned in a consistent manner. These procedures have remained unchanged throughout the study period, ensuring measurement reliability and inter-operator consistency.

Hormonal measurements

Serum follicle-stimulating hormone (FSH), luteinizing hormone (LH), prolactin (PRL), total testosterone (total T), free testosterone (free T), and estradiol (E2) were measured before any treatment. Reference ranges were based on institutional laboratory standards. Blood samples were obtained during routine clinic visits without a standardised collection time.

Statistical analysis

All statistical analyses were conducted using EZR software (Saitama Medical Centre, Jichi Medical University, ver. 1.68) and p-values <0.05 were considered to be significant. Differences between the two groups were examined using the Mann-Whitney U test.

## Results

Anthropometric characteristics

Clinical and anthropometric data are shown in Table 1. Thirty men with KS and 289 infertile men without KS were included in the analysis. Age and BMI did not significantly differ between the two groups. Patients with KS were significantly taller (median [interquartile range]: 174.5 [171.0-177.0] cm vs 171.0 [166.5-177.5] cm, p= 0.019), heavier (75.3 [62.7-90.9] kg vs 69.0 [61.5-76.3] kg, p = 0.017), and had a longer arm span (175.2 [170.9-180.0] cm vs 172.2 [167.0-177.5] cm, p = 0.046) than non-KS patients, consistent with eunuchoid body proportions.

**Table 1:** Anthropometric characteristics of infertile men with and without Klinefelter syndrome. * All values are expressed as the median (interquartile range). BMI: Body mass index

Variable*	KS (n = 30)	non-KS (n = 289)	p-value
Age (years)	35.5 (29.3-38)	34 (30-38)	0.66
Height (cm)	174.5 (171.0-177.0)	171.0 (166.5-177.5)	0.019
Weight (kg)	75.3 (62.7-90.9)	69.0 (61.5-76.3)	0.017
BMI (kg/m^2^)	24.5 (21.6-30.0)	23.6 (21.2-25.7)	0.10
Arm span (cm)	175.2 (170.9-180.0)	172.2 (167.0-177.5)	0.046

Hormonal characteristics

Hormonal characteristics are shown in Table 2. Patients with KS had markedly higher FSH and LH levels (both p <0.001) and significantly lower total and free T levels (both p <0.001) than patients without KS. PRL levels were lower in the KS group (p <0.001), whereas E2 levels did not significantly differ (p = 0.27).

**Table 2:** Hormonal characteristics of infertile men with and without Klinefelter syndrome. * All values are expressed as the median (interquartile range). Reference ranges: FSH 1.8-12.0 mIU/mL; LH 2.2-8.4 mIU/mL; PRL 4-14 ng/mL; Total T 131-871 ng/dL; Free T 4.6-23.8 pg/mL; E2 15-49 pg/mL. FSH: Follicle-stimulating hormone; LH: Luteinizing hormone; PRL: Prolactin; T: testosterone; E2: estradiol; KS: Klinefelter syndrome

Variable*	KS (n = 30)	non-KS (n = 289)	p-value
FSH (mIU/mL)	40.1 (23.5-47.5)	7.8 (4.4-17.9)	<0.001
LH (mIU/mL)	20.0 (14.0-23.5)	5.4 (3.8-7.9)	<0.001
PRL (ng/mL)	7.5 (6.0-10.0)	11.0 (8.0-15.0)	<0.001
Total T (ng/dL)	198.5 (124.5-295.8)	373.5 (284.8-492.0)	<0.001
Free T (pg/mL)	4.6 (2.9-5.6)	8.2 (6.2-10.5)	<0.001
E2 (pg/mL)	23.0 (17.0-27.8)	20.0 (14.0-26.0)	0.27

Summary

Overall, men with KS were taller and had a longer arm span, higher gonadotropin levels, and lower T levels than infertile men without KS.

## Discussion

In this single-centre cohort of Japanese men attending a male infertility clinic, patients with KS were taller, heavier, and-most notably a longer arm span than infertile men without KS, while BMI did not significantly differ. These anthropometric results align with the classical description of eunuchoid body proportions in KS and with clinic-based series reporting longer limb spans in a large percentage of patients [6,7]. These features are biologically plausible because androgen deficiency during puberty delays epiphyseal closure, allowing disproportionate limb elongation relative to trunk height [4,9].

The endocrine profile in our cohort-markedly elevated FSH and LH together with reduced total and free T-corroborates the canonical pattern of hypergonadotropic hypogonadism in KS described in previous series and comprehensive reviews [4,9,10]. E2 levels did not significantly differ between groups, which is consistent with findings showing that E2 values are often within the normal range in many KS men [6,7]. In contrast, PRL levels were significantly lower in the KS group (p < 0.001); however, median values in both groups remained within the normal reference range. Therefore, this difference is unlikely to be clinically meaningful and may reflect assay variations or subtle endocrine milieu differences rather than true hypoprolactinemia. Given that hormone assay platforms were updated during the 20-year study period, a small methodological contribution to PRL variability cannot be excluded.

From a clinical perspective, an arm span measurement is simple, non-invasive, and cost-free. Its feasibility and potential utility have been emphasised in infertility clinic cohorts that incorporated standardised anthropometry [6,7] and in contemporary guidelines recommending age-appropriate longitudinal follow-ups of growth and development in KS [11]. Despite these characteristic features, KS remains underdiagnosed, with many cases identified only in adolescence or adulthood [5,9,10]; therefore, pragmatic screening cues, such as an arm span assessment, may support earlier recognition in busy infertility clinics. Although the arm span/height ratio is also used clinically to assess eunuchoid body proportions, no standardised or evidence-based thresholds exist, and in our cohort, the ratio did not provide additional discriminatory value beyond absolute arm span.

The present study has several limitations. Its retrospective, single-centre design and modest KS sample-including a few mosaic cases, limits generalizability. Anthropometric ratios, such as the upper/lower segment ratio, were unavailable, and hormones were sampled once per patient; therefore, residual diurnal or assay variations cannot be excluded. In particular, testosterone levels were measured at varying times of day, and the lack of standardised morning sampling may have introduced additional diurnal variability. In addition, the study period exceeded 20 years because KS is a relatively uncommon condition, and adequate case accumulation required long-term inclusion. Although diagnostic awareness and referral patterns may have evolved over time, our institutional protocols for anthropometry and infertility evaluation remained largely unchanged. Thus, the impact of temporal variability on data uniformity is expected to be minimal. Future studies integrating anthropometric, hormonal, and metabolic parameters may further elucidate the in vivo endocrine mechanisms underlying KS phenotypes. Multicenter studies using harmonised anthropometric definitions and standardised outcome measures are warranted to validate these results and establish clinically useful reference values for KS screening in infertile men.

## Conclusions

Infertile Japanese men with KS had a longer arm span, higher gonadotropin levels, and lower testosterone levels than non-KS infertile men. These results, consistent with previous findings, support the presence of classical eunuchoid body proportions in Japanese populations and suggest that incorporating an arm span measurement may be a useful component of the routine evaluation of male infertility.
